# Biparatopic Protein Nanoparticles for the Precision Therapy of CXCR4^+^ Cancers

**DOI:** 10.3390/cancers13122929

**Published:** 2021-06-11

**Authors:** Olivia Cano-Garrido, Patricia Álamo, Laura Sánchez-García, Aïda Falgàs, Alejandro Sánchez-Chardi, Naroa Serna, Eloi Parladé, Ugutz Unzueta, Mònica Roldán, Eric Voltà-Durán, Isolda Casanova, Antonio Villaverde, Ramón Mangues, Esther Vázquez

**Affiliations:** 1 Nanoligent SL, Edifici EUREKA, Universitat Autònoma de Barcelona, Bellaterra, 08193 Barcelona, Spain; oliviacg@nanoligent.com (O.C.-G.); srnaroa@gmail.com (N.S.); 2 Institut de Biotecnologia i de Biomedicina, Universitat Autònoma de Barcelona, Bellaterra, 08193 Barcelona, Spain; laurasanchezgarcia92@gmail.com (L.S.-G.); Eloi.Parlade@uab.cat (E.P.); eric.volta@uab.cat (E.V.-D.); 3 CIBER de Bioingeniería, Biomateriales y Nanomedicina (CIBER-BBN), C/Monforte de Lemos 3-5, 28029 Madrid, Spain; PAlamo@santpau.cat (P.Á.); AFalgas@santpau.cat (A.F.); uunzueta@santpau.cat (U.U.); ICasanova@santpau.cat (I.C.); 4 Instituto de Investigación Biomédica Sant Pau (IIB Sant Pau), Sant Antoni Ma Claret 167, 08025 Barcelona, Spain; 5 Instituto de Investigación Contra la Leucemia Josep Carreras, 08025 Barcelona, Spain; 6 Departament de Genètica i de Microbiologia, Universitat Autònoma de Barcelona, Bellaterra, 08193 Barcelona, Spain; 7 Departament de Biologia Evolutiva, Ecologia i Ciències Ambientals, Facultat de Biologia, Universitat de Barcelona, Av. Diagonal 643, 08028 Barcelona, Spain; Alejandro.Sanchez.Chardi@uab.cat; 8 Servei de Microscòpia, Universitat Autònoma de Barcelona, Bellaterra, 08193 Barcelona, Spain; 9 Unitat de Microscòpia Confocal i Imatge Cel·lular, Servei de Medicina Genètica i Molecular, Institut Pediàtric de Malalties Rares (IPER), Hospital Sant Joan de Déu, Esplugues de Llobregat, 08950 Barcelona, Spain; mroldanm@sjdhospitalbarcelona.org; 10 Institut de Recerca Sant Joan de Déu, Esplugues de Llobregat, 08950 Barcelona, Spain

**Keywords:** tumor homing, EPI-X4, CXCR4, biparatopic nanoparticles, tumor targeting, drug delivery

## Abstract

**Simple Summary:**

Aimed at minimizing side toxicities cancer therapies require appropriate functional vehicles at the nanoscale, for receptor-mediated tumor-targeted drug delivery. The aim of the present study was to explore the human peptide EPI-X4 as a CXCR4-targeting agent in self-assembled, protein-only nanoparticles. While the systemic tumor biodistribution of EPI-X4-based materials is modest, this peptide shows potent proapoptotic effects on CXCR4^+^ cancer cells. Interestingly, the in vivo selectivity of EPI-X4 was dramatically improved, once combined into biparatopic nanoparticles, with a second CXCR4 ligand, the peptide T22. Biparatopic nanoparticles promote a highly selective tumor destruction in a mouse model of human colorectal cancer, probably associated to the CXCR4 antagonist role of EPI-X4. This study not only validates a new human ligand of the tumoral marker CXCR4, but it also offers a novel strategy for the combination, in protein nanoparticles, of dual acting ligands of tumoral markers for highly selective drug delivery.

**Abstract:**

The accumulated molecular knowledge about human cancer enables the identification of multiple cell surface markers as highly specific therapeutic targets. A proper tumor targeting could significantly avoid drug exposure of healthy cells, minimizing side effects, but it is also expected to increase the therapeutic index. Specifically, colorectal cancer has a particularly poor prognosis in late stages, being drug targeting an appropriate strategy to substantially improve the therapeutic efficacy. In this study, we have explored the potential of the human albumin-derived peptide, EPI-X4, as a suitable ligand to target colorectal cancer via the cell surface protein CXCR4, a chemokine receptor overexpressed in cancer stem cells. To explore the potential use of this ligand, self-assembling protein nanoparticles have been generated displaying an engineered EPI-X4 version, which conferred a modest CXCR4 targeting and fast and high level of cell apoptosis in tumor CXCR4^+^ cells, in vitro and in vivo. In addition, when EPI-X4-based building blocks are combined with biologically inert polypeptides containing the CXCR4 ligand T22, the resulting biparatopic nanoparticles show a dramatically improved biodistribution in mouse models of CXCR4^+^ human cancer, faster cell internalization and enhanced target cell death when compared to the version based on a single ligand. The generation of biparatopic materials opens exciting possibilities in oncotherapies based on high precision drug delivery based on the receptor CXCR4.

## 1. Introduction

A major challenge in cancer therapy is cell-targeted drug delivery intended to increase local drug concentration in tumor tissues, minimize systemic toxicities and improve the therapeutic outcome [1,2]. The molecular understanding of cancer-specific cellular processes and the identification of overexpressed surface antigens have rapidly expanded the number of therapeutic targets [3,4]. In this context, tumor cell targeting mediated by surface receptors is a smart strategy to enhance drug accumulation within pathogenic cells, taking advantage of the particular properties of drug carriers with nanoscale size [5,6,7]. Despite the progressive identification of specific receptor ligands by high-throughput techniques [8,9], there is still an important demand of biocompatible and efficient agents capable of promoting selective cell binding but also internalization of nanoparticles (NPs) and associated drugs, upon functionalization. The CXC chemokine receptor 4 (CXCR4) is a stem cell marker highly relevant in many solid and hematologic cancers, its overexpression being associated to dissemination [10] and poor prognosis [11,12]. Due to its significant role in cancer therapy, many ligands of this receptor have been identified. Most of the known CXCR4 antagonists are small molecules or cyclic peptides that cannot be produced in a recombinant form [13,14], and unable to penetrate cells via CXCR4-specific endocytosis. However, the horseshoe crab derivative CXCR4 ligand T22 [15] is one of the few tumor-homing peptides that shows high affinity for CXCR4 in a recombinant form, being also capable of promoting selective uptake of macro-molecular complexes and NPs inside CXCR4 overexpressing cells [16]. Then, its high potential as a precision ligand in the nanomedicine of cancer has been robustly supported, stressing its capability to selectively deliver conventional anticancer drugs [17], imaging agents [18] and proteins with cytotoxic activity, such as pro-apoptotic factors [19] and microbial, plant and animal toxins [20,21,22,23,24]. 

Recently, a novel human CXCR4 ligand has been described, namely the peptide EPI-X4, with relevance in physiological processes and diseases [25]. This peptide is generated by the proteolysis of the human serum albumin, under the acidic conditions that are characteristic of inflammatory processes and in tumor tissues [26]. Experimental performed in mouse models strongly suggest that EPI-X4 blocks CXCR4/CXCL12 cell signaling, thus suppressing the migration and invasion of cancer cells [25]. Regarding the potential clinical use of EPI-X4, its endogenous (human) origin assures lack of immunogenicity [27] and allows envisaging a broad therapeutic potential, in either mobilization of hematopoietic cells or as a cell targeting agent. In this context, we tested here the unexplored capacity of the human ligand EPI-X4 as a tumor-homing peptide in protein-based self-assembling NPs and their potential, together with T22, to form biparatopic agents aimed to enhance the specificity in targeting and internalization into CXCR4-overexpressing tumor cells. 

## 2. Materials and Methods

### 2.1. Protein Design, Production and Purification

Synthetic genes encoding modular proteins were designed in-house. The selected EPI-X4 sequence that was used for designing EPIX4-GFP-H6 and EPIX4-(RK)-GFP-H6, encoded the optimized dimeric version of the peptide with high affinity for CXCR4 [25]. In the case of EPIX4-(RK)-GFP-H6, a six cationic amino acid peptide (RKRKRK) was fused at the C-terminus of the EPI-X4 ligand to favor protein self-assembling as described elsewhere [28]. In addition, between EPI-X4 and the reporter GFP, a flexible peptidic linker (GGSSRSS) was added to favor ligand accessibility for CXCR4 binding. The gene codon usage was optimized for *E. coli* and the DNA segment was provided by Geneart (ThermoFisher) inserted into the plasmid pET22b (Novagen). The recombinant vector was transformed in *E. coli* BL21 (DE3) (F^–^ *ompT hsdSB* (rB^–^, mB^–^) *gal dcm* DE3) (Novagen). The encoded proteins were produced in Luria–Bertani (LB) media in 500 mL cell Erlenmeyer flasks at 20 °C overnight (O/N) upon addition of 0.1 mM Isopropyl β-d-1-thiogalactopyranoside (IPTG), when the OD_550_ of the cell culture reached around 0.5. Then, bacterial cells were harvested and centrifuged at 5000× *g*, for 15 min at 4 °C. The cell pellet was resuspended in wash buffer (20 mM Tris-HCl, 500 mM NaCl, 40 mM imidazole pH = 8) in presence of the protease inhibitor cocktail cOmplete EDTA-Free (Roche). Bacterial cells were disrupted in a French Press through three rounds at 1200 PSI, centrifuged (45 min, 15,000× *g*, 4 °C), and the soluble protein fraction purified by affinity chromatography with a HisTrap Chelating HP column in an AKTA purifier FPLC, (GE Healthcare). Upon sample filtration through a 0.22 µm pore filter and injection into the column, the protein was released from the column with elution buffer (20 mM Tris-HCl, 500 mM NaCl, 500 mM imidazol pH 8). Purified protein fractions were finally dialyzed against carbonate buffer (166 mM NaCO_3_H, pH 8). In addition, the CXCR4-targeted proteins T22-GFP-H6 and T22-BFP-H6 were produced and purified for the formation of biparatopic NPs as previously described [29].

### 2.2. Protein Characterization

The integrity of the recombinant proteins was checked by mass spectrometry (MALDI-TOF), TGX (Tris-Glycine eXtended, BioRad, Hercules, CA, USA) Stain-Free acrylamide gels electrophoresis (BioRad, Hercules, CA, USA) and Western Blot analysis using anti-His monoclonal antibody (1:1000; Santa Cruz, ref. 57598, Dallas, TX, USA). Protein concentration wasdetermined by Bradford (Biorad) assay with an albumin (Roche, Basel, Switzerland) standard curve. GFP fluorescence emission (510 nm) was determined on purified proteins with a Cary Eclipse fluorescence spectrophotometer (Agilent Technologies, Santa Clara, CA, USA) using an excitation wavelength of 450 nm. The volume and size distribution of NPs were measured by dynamic light scattering (DLS) at 633 nm through a Zetasizer Nano ZS (Malvern Instruments, Malvern, UK) using quartz cuvettes. 

### 2.3. Electron Microscopy 

Size and shape of protein nanoparticles at nearly state were evaluated with a field emission scanning electron microscope (FESEM). Protein samples were directly deposited over silicon wafers, excess of liquid blotted with Whatman filter paper, air-dried and observed without coating in a FESEM Zeiss Merlin (Zeiss, Oberkochen, Germany) operating at 1 kV and equipped with a high-resolution *in-lens* secondary electron detector. Representative images of nanoparticles were taken at a range of high magnifications (from 80,000× to 300,000×). 

### 2.4. Cell Culture, Flow Cytometry and Cytotoxicity Assay

Experiments were performed in CXCR4^+^ cervical and colorectal cell lines (HeLa and SW1417, respectively). HeLa cells were cultured in Eagle’s Minimum Essential Medium (Gibco, Waltham, MA, USA) and SW1417 in Dulbecco’s Modified Eagle’s Medium (Gibco). Both cell lines were supplemented with 10% fetal bovine serum (Gibco) and incubated in a humidified atmosphere at 37 °C and 5% (HeLa) or 10% (SW1417) CO_2_. For testing protein internalization, cells were seeded in 24-well plates (Nunc) (30000 cells per well) for 24 h. Briefly, the medium was removed, and cells were washed with PBS. Then, the protein, at 1 and 2 µM, was diluted in OptiPro medium supplemented with L-Glutamine and incubated at different times, at suitable cell line culture conditions. Then, harsh trypsin digestion (1 mg mL^−1^ for 15 min) (Gibco) was carried out to remove protein particles externally bound to cell membranes. Intracellular green fluorescence was analyzed by flow cytometry on a FACS-Calibur system (Becton Dickinson, Franklin Lakes, NJ, USA) using a 15 mW air-cooled argon ion laser at 488 nm excitation. Fluorescence emission was measured with a D detector (530/30 nm band pass filter), and manually corrected by the specific fluorescence of purified protein. This allowed getting data representative of the amount of internalized protein for comparative purposes. For competition assays, a specific CXCR4 antagonist AMD3100 (octahydrochloride hydrate, Sigma-Aldrich, San Luis, MO, USA) was added 1 h before NPs addition in a 1:10 (protein: AMD3100) molar ratio. Typically, we used 1 μM protein material exposed to cells for 1 h in conventional assays. For the analysis of EPIX4-GFP-H6, slightly higher protein concentration and extended incubation times were applied due to the poor uptake of this particular protein. All experiments were done in triplicate. 

### 2.5. Production and Characterization of Biparatopic Nanoparticles

T22-GFP-H6, T22-BFP-H6 and EPIX4-(RK)-GFP-H6 protein nanoparticles (at 1.5 mg ml^−1^) were disassembled by different methods. To T22-GFP-H6 and T22-BFP-H6 samples, NaCl (500 mM Na^+^ final concentration) and imidazole (300 mM final concentration) were added, and to EPIX4-(RK)-GFP-H6 samples we added 0.2% SDS, all of them into carbonate buffer (166 mM NaCO_3_H, pH 8). These mixtures were incubated for 2 h at room temperature (RT). T22-GFP-H6/EPIX4-(RK)-GFP-H6 and T22-BFP-H6/EPIX4-(RK)-GFP-H6 biparatopic NPs were generated by mixing the respective building blocks in a 1:1 molar ratio, and subsequently dialyzing them against carbonate buffer (166 mM NaCO_3_H, pH 8). We performed an exhaustive dialysis (4 changes every 30 min at RT, 1 change O/N at 4 °C and finally, 4 changes every 30 min). T22-BFP-H6/EPIX4-(RK)-GFP-H6 biparatopic NPs were used for FRET and confocal microscopy experiments and T22-GFP-H6/EPIX4-(RK)-GFP-H6 for FESEM, cell culture and in vivo experiments. To determine if this platform was capable to form heterogeneous NPs composed by both EPIX4-(RK)-GFP-H6 and T22-BFP-H6, FRET analysis was performed. Fluorescence emission of protein NPs was measured in a Cary Eclipse fluorescence spectrophotometer (Agilent Technologies) upon excitation at 387 nm. The emission was collected in the range from 400 to 600 nm. 

### 2.6. Cytotoxicity Studies 

The CellTiter-Glo^®^ Luminescent Cell Viability Assay (Promega) was used to determine the cytotoxicity of protein nanoparticles. SW1417 cells were plated in opaque-walled 96-well plates at 6000 cells per well in DMEM alpha medium supplemented with 10% foetal calf serum (Gibco) for 24 h at 37 °C until reaching 70% confluence. Then, cells were incubated in presence of several NP concentrations during 72 h at 37 °C. Subsequently, 100 µL of the single reagent (CellTiter-Glo^®^ Reagent, Promega Corporation, Madison, WI, USA) was added directly to cultured cells and the plates were measured in the Multilabel Plater Reader VICTOR3 (PerkinElmer, Waltham, MA, USA). Experiments were performed in triplicates. 

### 2.7. Analysis of Apoptosis

Specific apoptotic signaling was assessed, by the evaluation of positive cells for active cleaved caspase-3 as measured by IHC. Ki67 was also analyzed in tumor to search differences in tumor cell proliferation between groups. Both parameters were evaluated by two independent researchers, using an Olympus BX53 light microscope coupled to an Olympus DP73 digital camera.

### 2.8. Confocal Assay

HeLa cells were grown on Mat-Tek (MatTek Corporation, Ashland, MA, USA) plates (25,000 cells·wells^−1)^ in Eagle’s Minimum Essential Medium (Gibco) supplemented with 10% fetal bovine serum (Gibco) at 37 °C and 5% for 24 h. Then, 2 µM of protein NPs were added in OptiPro medium supplemented with L-Glutamine and incubated for 24 h at suitable cell culture conditions. Upon protein exposure, cell nuclei were labelled with 5 μg mL^−1^ Hoechst 33342 (Invitrogen, Waltham, MA, USA) and the plasma membrane with 2.5 μg mL^−1^ CellMask™ Deep Red (Molecular Probes, Eugene, OR, USA) for 10 min at room temperature. Confocal images were collected on an inverted TCS SP5 Leica Spectral confocal microscope (Leica Microsystems GmbH, Mannheim, Germany) using 63× (1.4 NA) oil immersion objective lenses. Excitation was reached via either a 405 nm blue diode laser (nucleic acids), a 488 nm line of an argon ion laser (NPs) or a 633 nm line of a HeNe laser (cell membrane). The confocal pinhole was set to 1 Airy unit and z-stacks acquisition intervals were selected to satisfy Nyquist sampling criteria. Confocal image stacks were reconstructed and visualized as three-dimensional (3D) volumes with Imaris software (Bitplane, Zurich, Switzerland).

### 2.9. Evaluation of EPIX4-(RK)-GFP-H6 and Biparatopic Nanoparticles Biodistribution in a Colorectal Subcutaneous Cancer Mouse

All in vivo experiments were approved by the institutional animal Ethics Committee of Hospital Sant Pau and by the Generalitat de Catalunya (protocol number 9721). We used a total of 36 five-week-old female Swiss Nu/Nu mice, weighing 18–20 g (Charles River), maintained in specific pathogen-free conditions. To generate the subcutaneous mouse model, we implanted subcutaneously 10 mg of the patient-derived M5 colorectal tumor tissue from donor animals in the mouse subcutis. When tumors reached approximately 500 mm^3^, mice received 200 μg single intravenous (i.v.) bolus of EPIX4-(RK)-GFP-H6 (*n* = 3 per time analyzed) or 200 μg single i.v. bolus of biparatopic NPs (*n* = 3 per time analyzed) in carbonate buffer (166 mM NaCO_3_H, pH 8). Control animals received the same buffer (*n* = 3 per time analyzed). At 0.5, 1, 2, 5 and 24 h mice were euthanized, and subcutaneous tumors and organs (brain, lung, liver, kidney and heart) were collected. Biodistribution of GFP NPs in tumor and non-tumor organs was determined by measuring the emitted fluorescence in ex vivo tissue sections (3 mm thick) using the IVIS^®^ Spectrum (Perkin Elmer) platform. The fluorescent signal (FLI), which correlates to the amount of administered protein accumulated in each tissue, was first digitalized, displayed as a pseudocolor overlay, and expressed as radiant efficiency [(p/sec/cm^2^/sr)/μW/cm^2^]. FLI values were calculated subtracting the FLI auto-fluorescence of control mice from the FLI signal of experimental mice. 

### 2.10. Histopathology and Detection of Apoptotic Bodies and Mitotic Figures

Samples were first fixed with 4% formaldehyde in PBS for 24 h to be embedded in paraffin, for histopathological evaluation and apoptotic index analyses. Number of apoptotic and mitotic figures were assessed in 4 μm sections of tumors and normal organs (liver, lung, spleen, heart, kidney and brain) stained with hematoxylin and eosin (H&E), which were histopathologically analyzed by two independent observers. Triton X-100 (0.5%) permeabilized sections were then stained with Hoechst 33258 (Invitrogen, Waltham, MA, USA) diluted, 1:5000 in PBS, for 1 h, rinsed with water, mounted and analyzed under fluorescence microscope (λex = 334 nm/λem = 465 nm). The number of mitotic figures and apoptotic bodies was quantified by recording cells in metaphase or condensed and/or defragmented nuclei per 10 high-power fields in H&E-stained tumor slices, respectively (magnification 400×), in blinded samples evaluated by two independent researchers.

### 2.11. Statistical Analyses

All quantitative values both of in vitro and in vivo experiments were expressed as mean ± standard error of mean ($\bar{x}$ ± SEM). Initially, overall differences among nanoparticles and effect of time were analyzed with Kruskal–Wallis and Friedman tests, respectively. Then, pairwise comparisons were made with Mann–Whitney tests. Differences among groups were considered significant at *p* < 0.05 and Bonferroni adjustment was applied for sequential comparisons. All statistical procedures were performed using SPSS 18 software (SPSS Inc., Chicago, IL, USA).

## 3. Results

**Figure 1:**
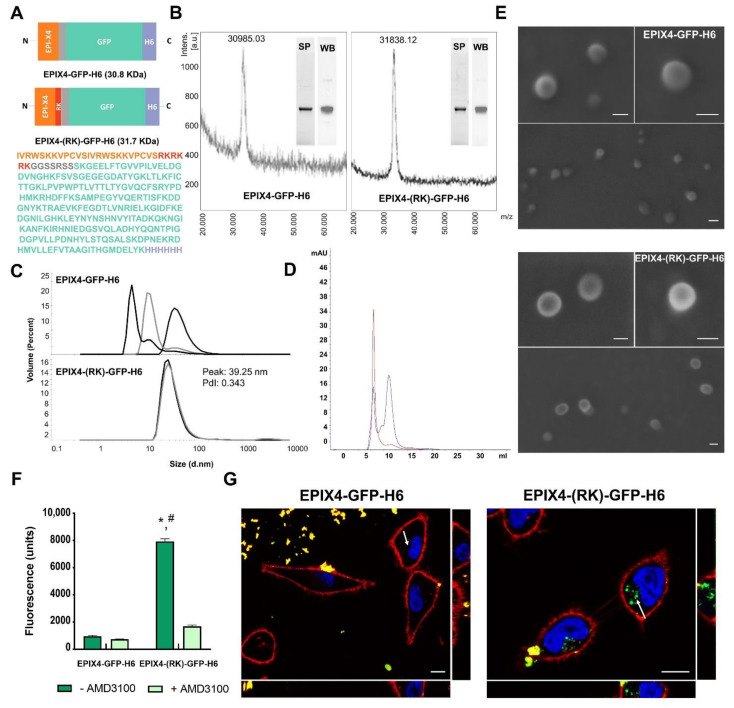
Structural and functional characterization NPs of EPI-X4-based. (**A**). Scheme of the modular protein EPIX4-GFP-H6 (top) and EPIX4-(RK)-GFP-H6 (bottom) and the full amino acid sequence of the bottom version. (**B**) Mass spectrometry of EPIX4-GFP-H6 (left) and EPIX4-(RK)-GFP-H6 (right). The purity of proteins is shown by SDS-PAGE (SP) and Western blot (WB) (Anti-His). (**C**) Hydrodynamic size and polydispersion index (PdI) of the protein materials determined by Dynamic Light Scattering (DLS). Values of peak size (mean) are indicated in nm. Each curve represents an individual measurement (*n* = 3). (**D**) Size exclusion chromatography (SEC) of EPIX4-GFP-H6 (blue) and EPIX4-(RK)-GFP-H6 (red) using a Superdex 200 increase 10/300GL column. (**E**) Representative FESEM (direct deposition) of EPIX4-GFP-H6 (top) and EPIX4-(RK)-GFP-H6 (bottom) protein NPs. Size bars represent 50 nm. (**F**) Protein NPs internalized into CXCR4^+^ HeLa cells after exposure to 2 µM EPIX4-GFP-H6 or EPIX4-(RK)-GFP-H6 for at 4 h (dark green) and visualized through their green fluorescence. Uptake inhibition mediated by the CXCR4 antagonist AMD3100 (light green). Intracellular fluorescence was corrected by their specific fluorescence to render values representative of protein amounts. The asterisk (*) indicates significant difference between internalization of EPIX4-GFP-H6 and EPIX4-(RK)-GFP-H6 (*p* = 0.021) and the hash (#) indicates significant difference between EPIX4-(RK)-GFP-H6 and the inhibition promoted by AMD3100 (*p* ≤ 0.001). (**G**) Confocal images of HeLa cells exposed to EPIX4-GFP-H6 (left) and EPIX4-(RK)-GFP-H6 (right) for 24 h. In blue: cell nuclei, in red: cell membrane, in green: internalized NPs. Size bars represent 10 µm. The right offset shows a Y-Z view and the bottom offset an X-Z view of the cell. The intracellular location of the protein material is indicated with a white arrow.

To explore the potential of EPI-X4 as a functional agent in tumor-targeted protein NPs usable as drug carriers in cancer, we performed a rational protein design to display EPI-X4 (Figure 1A) in self-assembling polypeptides. An optimized EPI-X4 tandem version with higher receptor affinity and serum stability [25] was genetically fused at the N-terminal of an H6-tagged GFP. As expected, the combination of a cationic peptide at amino terminus plus the polyhistidine (H6) carboxy terminal promoted protein assembling in NPs ranging from 10 to 80 nm in size, probably stabilized by divalent cation coordination through histidine-rich regions [30,31]. This size range is ideal for improving enhanced permeability and retention (EPR) effect and cell uptake, but also to minimize renal filtration (kidney cut-off around 6–8 nm) of any associated small molecular weight drugs [32]. Since only 25% of the EPI-X4 sequence contains cationic residues we engineered the protein to incorporate additional cationic amino acids (RKRKRK, named here as peptide RK) [28] to reach 50% of cationic residues in a new version of EPI-X4 (Figure 1A). 

Both protein versions (namely EPIX4-GFP-H6 and EPIX4-(RK)-GFP-H6) were efficiently produced in *Escherichia coli* and purified as pure full-length polypeptides with expected molecular masses (Figure 1B). While the parental version (EPIX4-GFP-H6) reached an unstable oligomerization in form of nanoparticulate entities of different sizes (from monomeric or dimeric forms of 4.8 and 8 nm to NPs of 10 and 50 nm, respectively), the protein version carrying the extra cationic sequence (EPIX4-(RK)-GFP-H6) spontaneously self-assembled as regular NPs of around 40 nm (Polydispersion Index, PdI = 0.343) (Figure 1C,D). In agreement, and fully supporting these results, FESEM examinations showed toroid (ring-shaped) materials with a regular morphometry (Figure 1E), that confirmed the measurements obtained by dynamic light scattering (DLS) and size-exclusion chromatography (SEC). When exposed to cultured CXCR4^+^ HeLa cells, only EPIX4-(RK)-GFP-H6 efficiently penetrated and accumulated intracellularly via CXCR4, as confirmed through the inhibition mediated by the CXCR4 antagonist AMD3100 [33] (Figure 1F). In addition, confocal images fully supported these data, showing the intracellular location of EPIX4-(RK)-GFP-H6 in absence of significant amounts of protein attached to the cell membrane (Figure 1G and Figure S1). 

**Figure 2:**
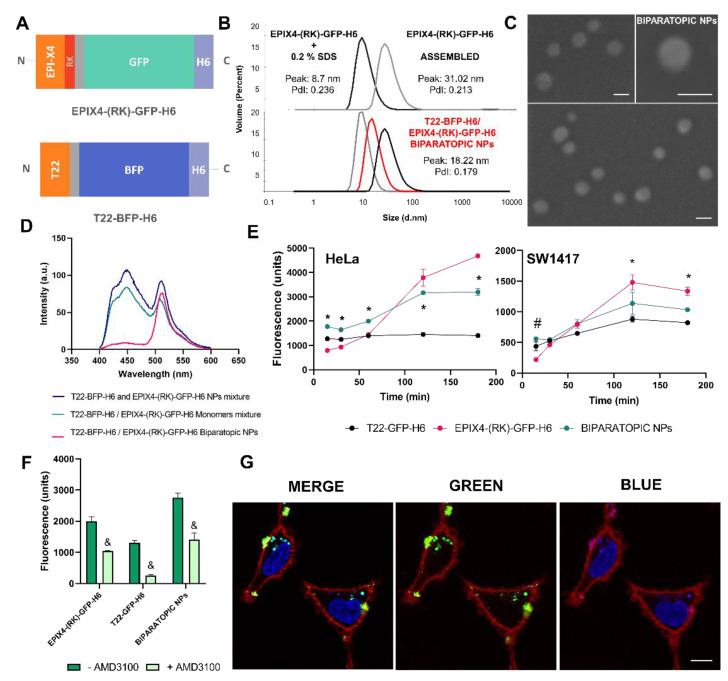
Formation and characterization of biparatopic NPs. (**A**) Scheme of the proteins EPIX4-(RK)-GFP-H6 (top) and T22-BFP-H6 (bottom) forming hybrid NPs. (**B**) Controlled EPIX4-(RK)-GFP-H6 disassembled by 0.2% SDS (black) and re-assembled removing SDS by dialysis (grey), determined by DLS (top). Hydrodynamic size comparison of T22-BFP-H6 (grey), EPIX4-(RK)-GFP-H6 (black) and biparatopic EPIX4-(RK)-GFP-H6/T22-BFP-H6 NPs (red) (bottom). Values of peak size (mean) are indicated (in nm) as well as PdI. (**C**) Representative FESEM images (direct deposition) of EPIX4-(RK)-GFP-H6/T22-BFP-H6 biparatopic NPs. Size bars represent 50 nm. (**D**) FRET analysis of biparatopic EPIX4-(RK)-GFP-H6/T22-BFP-H6 NPs. Samples of biparatopic NPs, T22-BFP-H6 and EPIX4-(RK)-GFP-H6 monomers mixture and T22-BFP-H6 and EPIX4-(RK)-GFP-H6 NPs mixture were excited with the 387 nm line and the emission was collected from 350–650 nm. BFP was used as donor fluorochrome and GFP as acceptor. (**E**) Time course kinetics of cell internalization of EPIX4-(RK)-GFP-H6, T22-GFP-H6 and biparatopic EPIX4-(RK)-GFP-H6/T22-GFP-H6 NPs (1 μM) in CXCR4^+^ HeLa cells (left) and SW1417 (right). Intracellular fluorescence was corrected by specific fluorescence to render values representative of protein amounts. Significant differences (*p* < 0.05) in the uptake values between biparatopic NPs and both forming proteins are depicted by and asterisk (*), and significant differences between biparatopic NPs and EPIX4-(RK)-GFP-H6 are depicted by a hash (#). (**F**) Uptake inhibition in HeLa cells exposed to 1 μM of EPIX4-(RK)-GFP-H6, T22-GFP-H6 or EPIX4-(RK)-GFP-H6/T22-GFP-H6 biparatopic protein material for 1 h, mediated by the CXCR4 antagonist AMD3100 (always at an excess molar ratio of 10:1). & indicates significant differences between the uptake of free NPs and upon inhibition by AMD3100 (*p* ≤ 0.001). (**G**) Confocal images of HeLa cells exposed to biparatopic EPIX4-(RK)-GFP-H6/T22-BFP-H6 NPs for 24 h, merged channels to show EPIX4-(RK)-GFP-H6 and T22-BFP-H6 protein colocalization (left) and green (middle) and blue channel (right). In blue: cell nuclei and T22-BFP-H6, in red: cell membrane, in green: EPIX4-(RK)-GFP-H6. Size bar represents 10 µm.

Recently, the capacity to control protein disassembling and reassembling in this histidine-based self-assembling protein platform has been demonstrated and the possibility to generate hybrid NPs made feasible [21,29,30,34]. The combination of different cell-ligands in the same construct is appealing in cancer therapy as it might dramatically increase cell specificity in drug delivery and also prevent the development of drug resistance [35,36]. In this context, we wanted to explore the structural versatility of our system to generate biparatopic NPs, able to bind CXCR4 through two different ligands, namely EPI-X4 and T22 (Figure 2A). First, we demonstrated that EPIX4-(RK)-GFP-H6 NPs could be disassembled to building blocks of 8.7 nm by using a mild detergent and reassembled by dialysis to form materials of the same size than the parental NP (Figure 2B). On this basis, we successfully mixed disassembled EPIX4-(RK)-GFP-H6 and T22-BFP-H6 (Figure 2A) to generate dual-color biparatopic NPs aimed to the in vitro characterization of the hybrid nanomaterial. In this sense EPIX4-(RK)-GFP-H6/T22-BFP-H6 biparatopic NPs resulted in a monodisperse population (PdI = 0.179) of about 18 nm (Figure 2B), morphologically indistinguishable (Figure 2C) from the original EPIX4-(RK)-GFP-H6 (Figure 1E) or from T22-BFP-H6 [29]. Additionally, the presence of both proteins in the same entity was corroborated by Förster resonance energy transfer (FRET) (from blue to green fluorescence), as determined by comparing fluorescence emission scans of different protein monomers against biparatopic NPs upon excitation at 387 nm. In this last case, when BFP is excited at 387 nm, the fluorescence emission energy of BFP is transferred to GFP chromophore, only observing GFP fluorescence emission at 510 nm (Figure 2D). 

On the other hand, to evaluate the biological properties of biparatopic NPs, single-color NPs were generated by successfully mixing disassembled EPIX4-(RK)-GFP-H6 and T22-GFP-H6 (structurally identical to T22-BFP-H6). This generated equivalent biparatopic NPs that allowed to perform comparative cell accumulation studies as both controls and hybrid materials can be measured at the same wavelength (ex: 488 nm). In this sense, CXCR4^+^ cells lines (the cervix cancer HeLa and the human colorectal SW1417) were exposed to these materials at different times. All NPs were internalized in both cell lines, being HeLa cells the line with higher uptake rate (*p* < 0.001). Although both cell lines express CXCR4, the expression levels are higher in HeLa (Figure S2), what might explain the better penetrability in this line. Those NPs keep the cell targeting properties (Figure 2E) without losing receptor specificity (Figure 2F). Cell internalization driven by T22 and monitored through T22-GFP-H6, showed high uptake levels at short times, while the uptake of EPIX4-(RK)-GFP-H6 occurred at longer times. Biparatopic EPIX4-(RK)-GFP-H6/T22-GFP-H6 NPs combined the uptake profile of both building blocks, being the cell uptake even significantly better at short times in both cell lines (Figure 2E). Confocal microscopy using dual-color biparatopic NPs confirmed the cell internalization and the co-localization of green and blue proteins supports the occurrence of true biparatopic NPs (Figure 2G and Figure S3). As previously described, biparatopic complexes might induce receptor clustering, which facilitates a faster cell internalization and lysosomal trafficking [37]. Selective targeting and rapid target cell penetrability could enhance the potential therapeutic effect of the material, thereby improving the specific entry in receptor positive tissues and avoiding off-target binding [36,38]. At the used range of protein concentration, none of these particles showed toxic effects on cultured CXCR4^+^ SW1417 cells upon exposure for 72 h (Figure S4).

**Figure 3:**
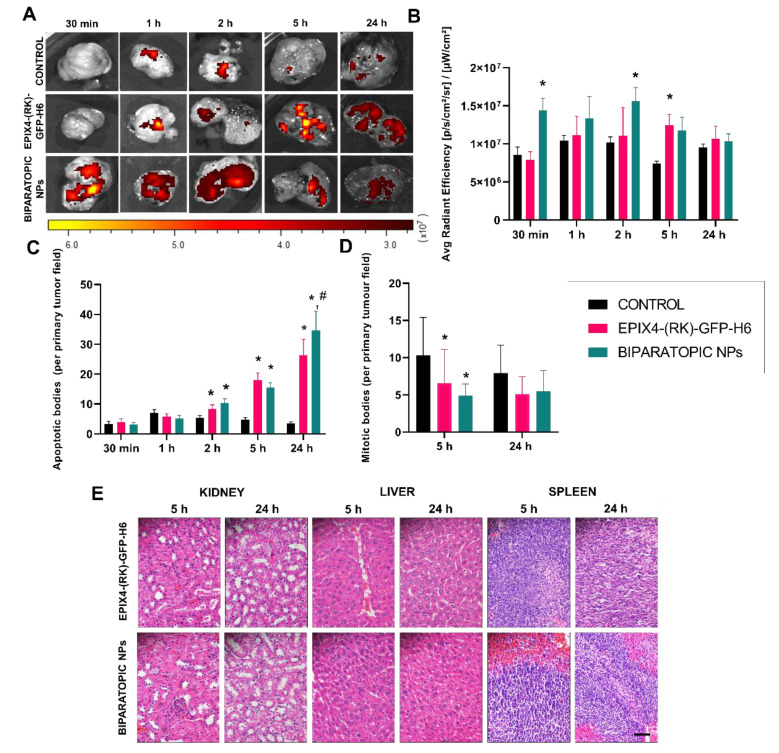
In vivo biodistribution, antitumor activity and systemic toxicity assessment in a subcutaneous mouse model of CXCR4^+^ human colorectal cancer. (**A**) FLI detection in tumors at different times after 200 μg single dose i.v. administration. Emission scales are shown as radiant efficiency units. Significant differences (*p* < 0.05) between EPIX4-(RK)-GFP-H6 or EPIX4-(RK)-GFP-H6/T22-GFP-H6 biparatopic NPs and the control are depicted by an asterisk (*). (**B**) Quantification of emitted fluorescence (measured as FLI ratio) at different times in tumors. (**C**) Number of apoptotic cell bodies upon NP administration. Significant differences (*p* < 0.05) between EPIX4-(RK)-GFP-H6 or biparatopic NPs and the control are depicted by an asterisk (*), while significant differences between biparatopic NPs and EPIX4-(RK)-GFP-H6 are depicted by a hash (#). A representation of the images source of these data are presented in Figure S5. (**D**) Number of mitotic figures after NP administration. Significant differences between EPIX4-(RK)-GFP-H6 and biparatopic NPs are depicted (* *p* < 0.05). (**E**) Lack of systemic toxicity in kidney, liver, kidney and spleen by histological analysis of tissue sections (H&E) 5 and 24 h after treatment. All pictures were taken at 400×. Size bar (bottom right) represents 50 µm.

At this stage, to assess in detail the clinical potential of targeted NPs, in vivo biodistribution analyses were performed in a CXCR4^+^ subcutaneous mouse model of patient-derived M5 colorectal cancer [21,39]. These mice models were treated with a single dose of 200 μg and fluorescence levels in tumor measured at different times (0.5, 1, 2, 5 and 24 h) and time significant patterns were found for the three NPs (*p* < 0.001). Single-color biparatopic NPs elicited a much faster tumor accumulation than EPIX4-(RK)-GFP-H6 NPs, reaching higher levels of intracellular material at shorter times (from 0.5 to 2 h) as predicted by in vitro analysis (Figure 2E). This could be due to the participation in the complexes of T22 that has been proved to be an excellent CXCR4 ligand in vivo, promoting an unusually precise biodistribution of drug carriers and protein-drug nanoconjugates in cancer models [17,18]. It must be noted that such biodistribution is receptor-specific and mediated by the T22 peptide ligand, since similar nanoparticles functionalized with an N-terminal R9 peptide did not show accumulation in CXCR4^+^ tumor tissues in the same animal model [18]. Additionally, in vivo tumor accumulation of T22-empowered NPs is efficiently inhibited by the CXCR4 antagonist AMD3100 [40]. On the other hand, EPIX4-(RK)-GFP-H6 showed a progressive accumulation that peaked at 5 h, reaching levels that remained relatively stable at least for 24 h (Figure 3A,B). Interestingly, in those target tissues, we detected a significant increase of apoptotic bodies (Figure 3C and Figure S5A) and a reduction of mitotic figures (Figure 3D), indicative of a cytotoxic effect of the material. Apoptosis was confirmed by the activation of caspase 3 (Figure S5B). When analyzing normal tissues, fluorescence was low in non-tumoral tissues for all nanoparticle versions. Interestingly, the low fluorescence levels in kidney at longer times was an indicator that this hetero-oligomeric platform retains the nanoparticle stability in vivo as the parental version does (Table 1). In addition, histopathological analyses indicated the lack of systemic toxicity neither in CXCR4^−^ tissues (kidney and liver) or CXCR4^+^ (spleen) at the cellular level (Figure 3E). This fact strongly suggests that the fluorescence levels observed in these tissues might correspond to circulating material rather than to internalized NPs. Additionally, this data also supports the selectivity of tissue destruction shown in Figure 3C,D.

The CXCR4 receptor blockade has been deeply described as a way to induce cell apoptosis [41,42,43]. In this regard, the potential presence of apoptotic cell bodies induced by the EPI-X4-based constructs was analyzed by histological evaluation. Throughout time, it was observed a dramatic increase of apoptotic events of these constructs that was significant for EPI-X4 NPs (*p* = 0.002) but was more pronounced when administering the biparatopic NPs (*p* < 0.001), with a drop in the number of mitotic cells (Figure S5A) in the tumor samples, being both significant at 5 h (Figure 3D). The more efficient triggering of cell death in the biparatopic materials could be due to their faster uptake of that provoked higher levels of receptor internalization than the other material version, only displaying EPI-X4. This fact elicits a substantially higher number of apoptotic bodies at 24 h (Figure 3C and Figure S5A,B). Altogether, these data strongly suggest that EPI-X4 might induce apoptosis through its interaction with CXCR4.

## 4. Discussion

Taken together and specially looking at data in Figure 3A, all the collected observations indicate the moderate potential of EPI-X4 as a human tumor-homing peptide. However, a proper protein engineering together with the combination of ligands in hybrid materials resulted into regular and stable protein NPs with a potent CXCR4-targeting, whose specificity for in vivo biodistribution was enhanced by the complementation with the additional CXCR4 binder T22. Indeed, the plasticity of this type of protein material permitted such combination as structurally robust biparatopic NPs, with high penetrability at short circulation times in blood and enhancing the parental specificity and biodistribution pattern. Regarding the antitumor activity of the biparatopic NPs, our results suggest that the multivalent display of the ligands EPI-X4 and T22, which most likely interact with different CXCR4 domains, are probably responsible for the faster rate of internalization and the enhanced induction of apoptosis in the biparatopic setting. Thus, the peak of around 30 apoptotic figures per 400 x magnification tumor field, reached 24 h post-treatment, is of a similar magnitude than the induction of cell death in previous findings. These were based on colorectal tumor models after treatment with NPs, that based on T22, incorporate potent cytotoxic agents such as Floxuridine [17] or the catalytic domain of bacterial toxins [21,44,45]. Therefore, in the biparatopic setting, T22 could assist by enhancing targeting and specially the cell penetrability mediated by EPI-X4 and therefore, their pro-apoptotic effects. In the hybrid materials, EPI-X4 appears to be the only ligand responsible for promoting cell death as an efficient CXCR4 antagonist. In this regard, it must be noted that T22, in form of different types of protein-only nanoparticles (including GFP, BFP or iRFP) has been never observed as inducer of cell toxicity or apoptosis in cell culture or in vivo, in different animal models of human cancers [16,46,47,48,49,50,51]. However, the use of T22-based vehicles to deliver small cytotoxic drugs [17,52,53] or proteins [24,40,45,48,54] result in highly selective tumor tissue destruction. These consistent observations confirm that in the biparatopic materials EPI-X4 is associated to cell toxicity while T22 to enhanced targeting. 

One of the main goals in nanotechnology is the development of materials and vehicles which permit extending the drug half-life and to enhance bioavailability [55], accompanied by precise tumor targeting in drug release, to reduce therapeutic doses and to avoid side toxicity in health tissues [56]. Protein-based nanomaterials show appealing therapeutic applications given their unique properties, that include full biocompatibility, degradability, structural and functional versatility and ease of production, apart from self-assembling properties [57,58,59,60,61]. The principal criteria of the regulatory agencies to approve a new drug are related to efficacy and safety. Then, drug cell-targeting is the best approximation to deliver higher doses of therapeutic agents without affecting healthy tissues [38]. Currently, there are a few antibody-drug conjugates in the market in which targeting permits to increase the reported maximum tolerated dose (MTD) around 10 times over than that of the free drug [62,63,64]. These new products have recently opened an opportunity to develop novel targeted products for the clinic. Discovering new ligands that exclusively recognize a specific tumor target and that are suited for a multivalent presentation is now challenging to develop non-antibody carriers for conventional and unconventional drugs. Among all the existing agents, peptides are particularly appealing because of their short size and cost-effective production processes [65,66].

A wide variety of CXCR4 specific binders have been described, such as the natural ligand CXCL12 [67,68], gp120 and V3 (from HIV) [69], vCCL2 (from herpes virus) [70], T22 (from horseshoe carbs) [16] and synthetic polypeptides (CGPG422 and R9) [71,72]. The use of non-human sequences could trigger immunogenic responses influencing the potential drug safety and pharmacokinetics [41]. Although CXCL12 presented appealing results in receptor binding [67] their nature as endogenous agonist of the CXCR4 receptor prevents its use in therapy because it activates CXCR4 downstream signaling. In contrast, EPI-X4 as endogenous antagonist, being not useful as tumor-homing peptide, is highly efficient in suppressing signaling from CXCR4 and in inhibiting CXCL12-induced cancer cell migration in vitro and inflammatory cell infiltration in vivo [25]. In this context, the data presented here reveal the proapoptotic and antimitotic effects of EPI-X4 (Figure 3C,D), that can be selectively targeted with the assistance of a properly working tumor homing peptide such as T22. Interestingly, such cytotoxic activities are not observed in cell culture upon moderate exposure times (Figure S4). In our hands, other pro-apoptotic peptides or protein domains from different origins including PUMA, BAXPORO, GWH1 and BAK, show no cytotoxic effects in cell culture but significant induction of apoptosis and reduction in the number of mitotic cells in vivo [40,46,73]. This contrast between the lack of antitumor activity in vitro and the potent induction of apoptosis in cancer cells in vivo may be based on the different regulation of these cells by the cell culture media as compared to their regulation by tumor microenvironment. Monolayer cultured cells do not mimic tumor cell-extracellular environment interactions affecting morphology, polarity, differentiation, proliferation and cell death, and particularly regulation of apoptosis [74]. Besides, the unlimited access to medium, oxygen or nutrients and the generated metabolites or activated signaling in vitro differs from the situation in the scarcity of oxygen or nutrients in fast growing tumors. In contrast, the tumor microenvironment provides niches that protect and maintain cancer stem cells (CSCs) [75]. In this regard, the maintenance of CXCR4^+^ CSCs depends on its natural signaling ligand SDF1α. Then, their interaction with stromal or nurse-like cells capable of secreting SDF1α in several cancer types provide a protective niche where they are quiescent, an aspect that is clearly different from the exponential growth in cell culture [76,77]. Consequently, in vivo, the CXCR4 peptide antagonist TN14003 displaces CXCR4^+^ cancer cells from their niche, rendering them sensitive to apoptotic induction, an observation that could be replicated in vitro when cancer cells are co-cultured with SDF1α-expressing stromal cells [78]. 

Finally, it is worth mentioning the interest to explore in the future if EPI-X4, similarly to AMD3100 [79], might also mobilize hematopoietic precursors from the bone marrow, a non-unexpected property that might add therapeutic value to this pleiotropic peptide. 

## 5. Conclusions

The albumin-derived ligand EPI-X4 promotes selective internalization of self-assembling protein-only nanoparticles into CXCR4^+^ cells. In addition, this agent, as a potent CXCR4 antagonist, shows relevant pro-apoptotic effects in tumor tissues in a mouse model of human colorectal cancer. Upon systemic administration, the tumor biodistribution of EPI-X4-empowered NPs is largely improved by an additional CXCR4 ligand, the peptide T22, organized with EPI-X4 in form of multivalent biparatopic nanoparticles. The exploration of EPI-X4 as a ligand of the tumoral marker CXCR4 allows the exploitation of a novel humanized tool, and it also offers realistic possibilities for the development of a new generation of safe and stable drugs for targeted cancer therapy.

## 6. Patents

A patent has been granted for the use of T22 and EPI-X4 as targeting agents in cancer treatments (WO2012095527).

## Figures and Tables

**Table 1:** Tissue biodistribution of CXCR4-targeted protein nanoparticles upon systemic administration. Fluorescence emitted by normal organs (liver, kidney, spleen, lung, bone marrow and brain) at the analyzed times (0.5, 1, 2, 5 and 24 h) after the administration of 200 μg of EPIX4-(RK)-GFP-H6, EPIX4-(RK)-GFP-H6/T22-GFP-H6 biparatopic NPs or carbonate buffer as a control, expressed as $\bar{x}$ ± SEM of radiant efficiency [× 10^6^ (p/sec/cm^2^/sr)/μW/cm^2^]. ^a^ *p* = 0.021, ^b^ *p* = 0.011 (Mann–Whitney test).

	LIVER	KIDNEY	SPLEEN	LUNG	BONE MARROW	BRAIN
	**0.5 h**					
**BUFFER**	4.59 ± 0.9	3.24 ± 0.1 ^a,b^	3.14 ± 0.4	2.5 ± 0.1	4.0 ± 0.2	3.0 ± 0.2
**EPIX4-(RK)-GFP-H6**	7.14 ± 0.6	7.14 ± 0.7 ^a^	4.2 ± 0.5	5.0 ± 0.5	5.1 ± 0.4	4.1 ± 0.1
**BIPARATOPIC NPs**	7.02 ± 0.5	7.02 ± 0.8 ^b^	4.5 ± 0.4	3.8 ± 0.3	4.3 ± 0.6	3.4 ± 0.4
	**1 h**					
**BUFFER**	4.35 ± 0.0	3.62 ± 0.7	2.7 ± 0.0	3.0 ± 0.0	4.1 ± 0.0	3.6 ± 0.0
**EPIX4-(RK)-GFP-H6**	4.93 ± 0.3	4.93 ± 0.4	3.6 ± 0.2	3.3 ± 0.2	5.0 ± 0.1	3.9 ± 0.1
**BIPARATOPIC NPs**	4.73 ± 0.2	4.73 ± 1.1	3.3 ± 0.2	2.7 ± 0.5	4.1 ± 0.9	3.4 ± 0.2
	**2 h**					
**BUFFER**	4.57 ± 0.2	3.73 ± 0.3	3.4 ± 0.2	2.9 ± 0.3	5.2 ± 0.04	3.4 ± 0.4
**EPIX4-(RK)-GFP-H6**	4.46 ± 0.1	4.46 ± 0.4	4.4 ± 0.3	3.5 ± 0.5	5.3 ± 0.9	4.3 ± 1.0
**BIPARATOPIC NPs**	4.64 ± 0.4	4.64 ± 0.5	3.7 ± 0.2	2.8 ± 0.1	4.1 ± 0.2	4.0 ± 0.3
	**5 h**					
**BUFFER**	4.78 ± 0.3	4.3 ± 0.4	3.5 ± 0.3	2.7 ± 0.3	4.5 ± 0.1	3.7 ± 0.2
**EPIX4-(RK)-GFP-H6**	4.53 ± 0.2	4.53 ± 2.2	4.3 ± 0.2	2.9 ± 0.1	4.3 ± 0.2	4.7 ± 0.9
**BIPARATOPIC NPs**	3.93 ± 0.3	3.93 ± 1.9	3.9 ± 0.5	2.5 ± 0.1	4.5 ± 0.3	3.6 ± 0.2
	**24 h**					
**BUFFER**	4.48 ± 1.3	4.48 ± 1.8	4.4 ± 0.4	2.6 ± 0.2	5.1 ± 0.4	3.5 ± 0.2
**EPIX4-(RK)-GFP-H6**	5.36 ± 0.2	5.36 ± 2.2	4.5 ± 0.4	2.6 ± 0.1	4.8 ± 0.1	5.3 ± 0.8
**BIPARATOPIC NPs**	4.38 ± 2.0	4.38 ± 2.2	4.0 ± 0.2	2.8 ± 0.1	4.6 ± 0.3	4.8 ± 0.6

## Data Availability

Original quantitative data from the experimental can be found at https://doi.org/10.5565/ddd.uab.cat/240829 (accessed on 9 June 2021).

## Supplementary Materials

The following are available online at https://www.mdpi.com/article/10.3390/cancers13122929/s1, Figure S1. CXCR4 cell internalization of EPIX4-(RK)-GFP-H6 and EPIX4-GFP-H6 nanoparticles; Figure S2. CXCR4 expression levels in HeLa and SW1417 cell lines; Figure S3. T22-BFP-H6 and EPIX4-(RK)-GFP-H6 colocalization in biparatopic nanoparticles; Figure S4. Viability of CXCR4^+^ SW1417 cells upon exposure to protein nanoparticles. Figure S5. Tumor cell death in vivo promoted by protein NPs.
