# Supplementary material for: Biparatopic Protein Nanoparticles for the Precision Therapy of CXCR4+ Cancers
Source: Cancers (Basel). 2021 Jun 11;13(12):2929. doi: 10.3390/cancers13122929 (PMC8230831; doi:10.3390/cancers13122929)
Supplement: Supplementary file 1 [file cancers-13-02929-s001.zip › cancers-1198403-supplementary.pdf]

cancers

MDPI

Supplementary Files

Biparatopic Protein Nanoparticles for the Precision Therapy of CXCR4+ Cancers

Olivia Cano-Garrido, Patricia Álamo, Laura Sánchez-García, Aïda Falgàs, Alejandro Sánchez-Chardi, Naroa Serna Eloi Parladé, Ugutz Unzueta, Mònica Roldán, Eric Voltà-Durán, Isolda Casanova, Antonio Villaverde, Ramón Mangues and Esther Vázquez

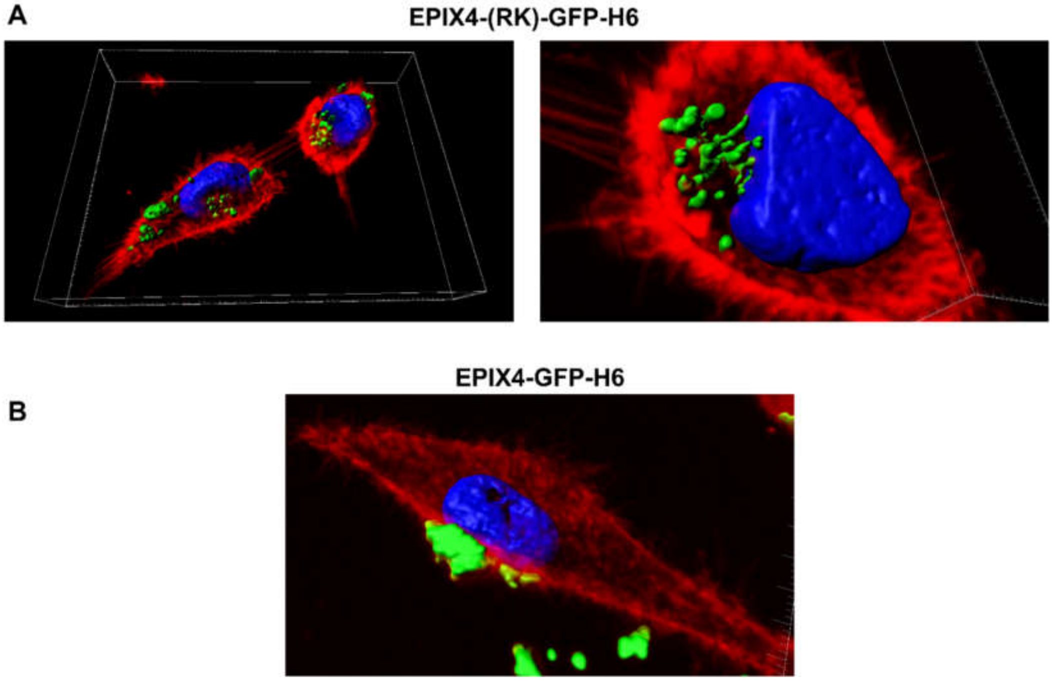

Figure S1. CXCR4 cell internalization of EPIX4-(RK)-GFP-H6 and EPIX4-GFP-H6 nanoparticles. 3D reconstructions of Figure 1G. HeLa cells exposed to 2 µM EPIX4-(RK)-GFP-H6 (**A**) and EPIX4-GFP-H6 (**B**) for 24 h. In blue: cell nuclei, in red: cell membrane, in green: NPs. Scale bar, A: 5µm, A in detail: 3 µm, B: 5µm.

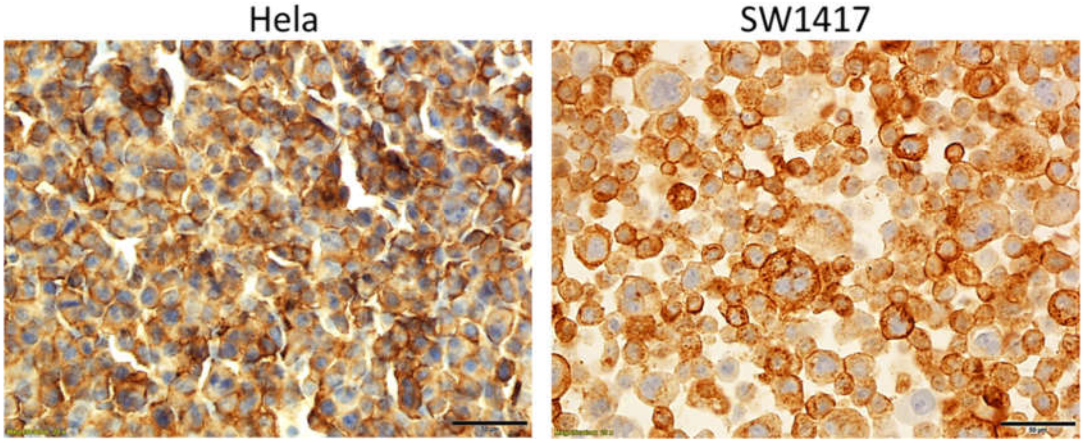

Figure S2. Immunohistochemistry of CXCR4 in cultured HeLa and SW1417 cell lines. Magnification is 400 × and the size bars indicate 50 µm.

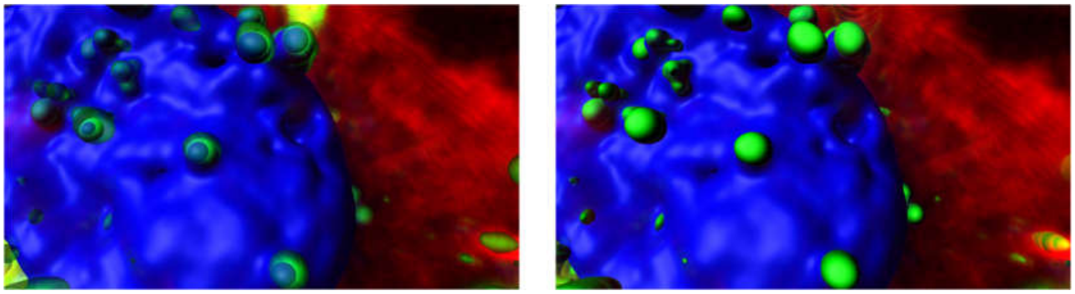

Figure S3. T22-BFP-H6 and EPIX4-(RK)-GFP-H6 colocalization in biparatopic nanoparticles. 3D reconstructions of Figure 2G. HeLa cells exposed to 2 μM T22-BFP-H6 (left) and EPIX4-(RK)-GFP-H6 (right) for 24 h. In blue: cell nuclei and T22-BFP-H6 NPs, in red: cell membrane, in green: EPIX4-(RK)-GFP-H6 NPs.

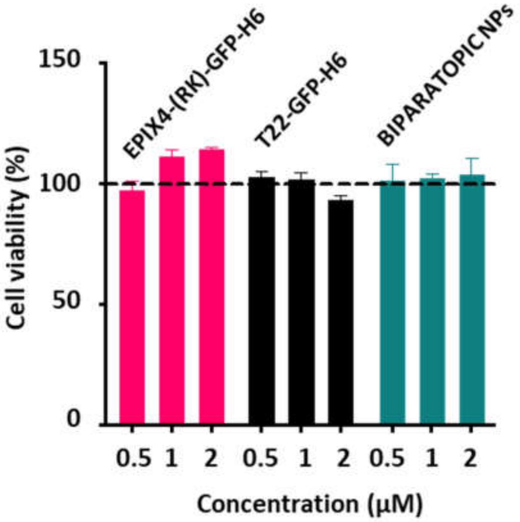

Figure S4. Viability of $CXCR4^{+}$ SW1417 cells upon exposure to protein nanoparticles. Incubation time was 72 h, and cell viability was determined by a conventional luminescent cell viability assay. No significant differences between proteins and used concentrations were observed.

(**A**)

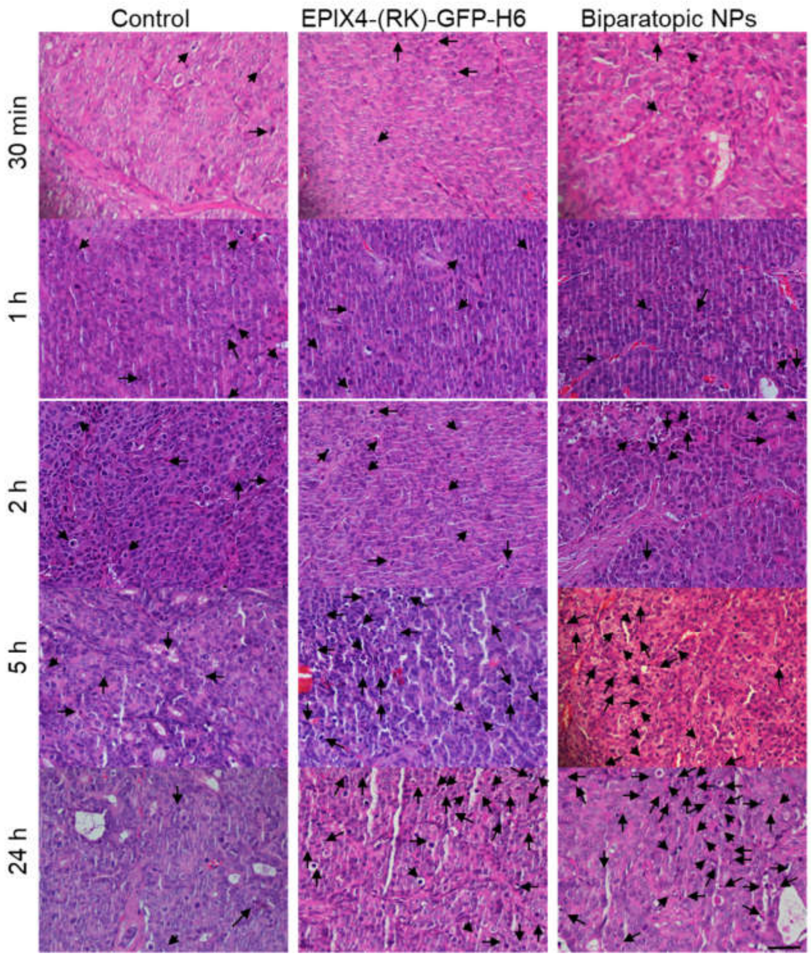

(**B**)

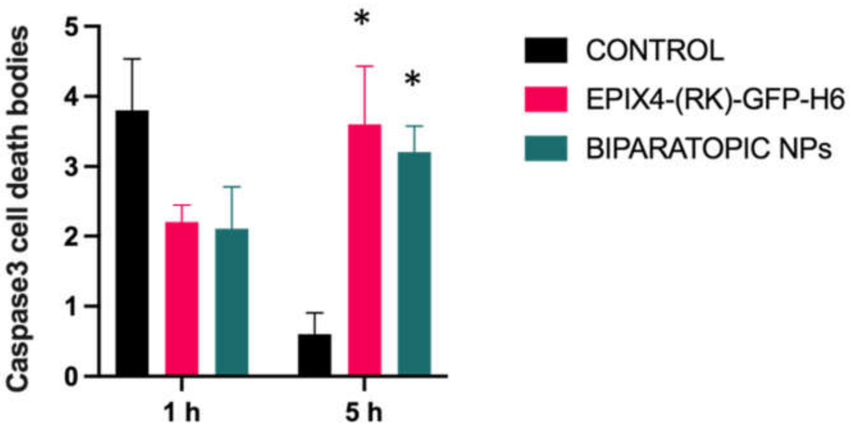

Figure S5. Tumor cell death in vivo promoted by protein NPs. (**A**) Cell death bodies (indicated by arrows) observed in tumoral tissues upon systemic administration of protein NPs. Quantitative data are shown in Figure 3C. Size bar at the bottom left represents 50 µm. (**B)** Caspase 3 activation in tumoral tissues determined at two different times upon administration of protein NPs. Significant differences between EPIX4-(RK)-GFP-H6 (p=0.001) or biparatopic (p = 0.003) NPs and control NPs (T22-GFP-H6) are depicted by an asterisck (*).
